# Macrophage Cytokines: Involvement in Immunity and Infectious Diseases

**DOI:** 10.3389/fimmu.2014.00491

**Published:** 2014-10-07

**Authors:** Guillermo Arango Duque, Albert Descoteaux

**Affiliations:** ^1^INRS-Institut Armand-Frappier, Laval, QC, Canada; ^2^Centre for Host-Parasite Interactions, Laval, QC, Canada

**Keywords:** macrophage, cytokine, trafficking, exocytosis, proinflammatory, anti-inflammatory, *Leishmania*, *Mycobacterium ulcerans*

## Abstract

The evolution of macrophages has made them primordial for both development and immunity. Their functions range from the shaping of body plans to the ingestion and elimination of apoptotic cells and pathogens. Cytokines are small soluble proteins that confer instructions and mediate communication among immune and non-immune cells. A portfolio of cytokines is central to the role of macrophages as sentries of the innate immune system that mediate the transition from innate to adaptive immunity. In concert with other mediators, cytokines bias the fate of macrophages into a spectrum of inflammation-promoting “classically activated,” to anti-inflammatory or “alternatively activated” macrophages. Deregulated cytokine secretion is implicated in several disease states ranging from chronic inflammation to allergy. Macrophages release cytokines via a series of beautifully orchestrated pathways that are spatiotemporally regulated. At the molecular level, these exocytic cytokine secretion pathways are coordinated by multi-protein complexes that guide cytokines from their point of synthesis to their ports of exit into the extracellular milieu. These trafficking proteins, many of which were discovered in yeast and commemorated in the 2013 Nobel Prize in Physiology or Medicine, coordinate the organelle fusion steps that are responsible for cytokine release. This review discusses the functions of cytokines secreted by macrophages, and summarizes what is known about their release mechanisms. This information will be used to delve into how selected pathogens subvert cytokine release for their own survival.

## Introduction: Cytokines and Macrophages

Macrophages are phagocytic cells of the innate immune system that are located in various tissues. The Russian scientist Elie Metchnikoff received the 1908 Nobel Prize in Physiology or Medicine for his work on immunity when he observed that when he punctured starfish larvae, a population of cells migrated to the wound. He also observed cells that were able to uptake particles that had been placed in the digestive tracts of the larvae. Elie Metchnikoff coined these cells as phagocytes and later called them white blood cells for their first-line-of-defense role against infection in living organisms (1). Later, the term macrophage was introduced by Aschoff in 1924 to designate a set of cells of the reticuloendothelial system formed not only by monocytes, macrophages, and histiocytes, but also by fibroblasts, endothelial, and reticular cells. After 1969, the concept of the mononuclear phagocyte system – formed by a variety of macrophages derived from monocytes from the bone marrow – was introduced to replace the concept of the reticuloendothelial system, which is constituted of functionally and immunologically distinct cells. Most macrophages are derived from bone marrow precursor cells that develop into monocytes. These are formed in the bone marrow from stem cells of the granulocytic–monocytic lineage that are exposed to cytokines such as the granulocyte macrophage colony stimulating factor (GM-CSF) and interleukin-3 (IL-3). Differentiation from stem cells is associated with the expression of specific membrane receptors for cytokines. Monocytes remain in the bone marrow <24 h and they move into the bloodstream and circulate throughout the body. In normal healthy adults, the half-life of a circulating monocyte is estimated at 70 h. Monocytes constitute 1–6% of total leukocytes in healthy peripheral blood. After crossing the walls of capillaries into connective tissue, monocytes turn into macrophages. This differentiation process involves many changes as the cell increases in size from 5 to 10 times, its organelles increase both in number and complexity, phagocytic capacity increases, etc. It is important to note that not all macrophages, such as Langerhans cells and brain microglia, develop from monocytes (2).

The main function of macrophages is to engulf foreign agents that enter the body. These include microbes and other particulate matter. In addition, they eliminate apoptotic cells and recycle nutrients by digesting waste products from tissues. Macrophages are hence essential not only for immunity, but also for development and tissue homeostasis (2). These cells are normally at rest, but can be activated by a variety of stimuli during the immune response (3, 4). Albeit phagocytosis may provide the initial antigen stimulus, the activity of macrophages can be increased by cytokines secreted by helper T cells, with interferon gamma (IFN-γ) being one of the most potent macrophage activators. In addition, these multifaceted cells are also capable of chemotaxis, namely the process of being attracted and displaced to a particular location by specific molecules. Besides phagocytosis, macrophages play a central role in inflammation. They initiate the immune response against microorganisms, since macrophages are some of the first cells to come in contact with these invaders. This is in part due to their toll-like and scavenger receptors, which have broad ligand specificity for lectins, lipoproteins, proteins, oligonucleotides, polysaccharides, and other molecules. In addition to these functions, macrophages express major histocompatibility complex (MHC) class II molecules on their membranes, and as such, also present antigens to lymphocytes. When macrophages engulf a microbe, its antigens are processed and situated on the outer surface of the plasmalemma, where they will be recognized by T helper cells. Following this recognition, T lymphocytes release cytokines that activate B cells, and activated B lymphocytes then secrete antibodies specific to the antigens presented by the macrophage. These antibodies attach to antigens on microbes, or to cells invaded by microbes; in turn, these antibody-bound complexes are phagocytosed more avidly by macrophages.

Cytokines and chemokines are potent signaling molecules that are as important to life as hormones and neurotransmitters. They are low molecular weight proteins that mediate intercellular communication and are produced by many cell types, primarily those of the immune system. They were discovered in the early 60–70s, and today, over 100 different proteins are known as cytokines (5). These molecules orchestrate a variety of processes ranging from the regulation of local and systemic inflammation to cellular proliferation, metabolism, chemotaxis, and tissue repair. In other organisms, such as fruit flies and lizards (6), cytokine-like molecules are known to regulate host defense and temperature homeostasis. The primary function of cytokines is to regulate inflammation, and as such, play a vital role in regulating the immune response in health and disease. There are proinflammatory and anti-inflammatory cytokines.

Each cytokine binds to a specific cell surface receptor to generate a cell signaling cascade that affects cell function. This includes the positive or negative regulation of several genes and their transcription factors. This may ensue in the production of other cytokines, in an increase in the number of surface receptors for other molecules, or eventually in the suppression of the cytokine’s own effect. Each cytokine is produced by a cell population in response to different stimuli; they induce an array of agonist, synergistic, or antagonistic effects that functionally alter target cells. A primary feature of cytokine biology is that of functional redundancy: different cytokines share similar functions. Furthermore, cytokines are pleiotropic since they act on many different cell types, and cells may express more than one receptor for a given cytokine. To generalize the effect of a particular cytokine is virtually impossible. Cytokines are classified as paracrine if the action in the vicinity of the place of release is restricted, autocrine if the cytokine acts on the cell that secretes it, and endocrine if the cytokine reaches remote regions of the body. Most cytokines are short-lived and act locally in an autocrine and paracrine fashion. Only some cytokines present in the blood, such as erythropoietin (EPO), transforming growth factor beta (TGF-β), and monocyte colony stimulating factor (M-CSF), are capable of acting at a distance.

Cytokines are mainly produced by macrophages and lymphocytes, although they can also be produced by polymorphonuclear leukocytes (PMN), endothelial and epithelial cells, adipocytes, and connective tissue. Cytokines are essential to the functions of macrophages. They mediate the unleashing of an effective immune response, link innate and adaptive immunity, and influence the macrophage’s microenvironment (4, 7). Multiple subsets of macrophages have been characterized depending on the origin and microenvironment in which the macrophage is found. Contingent on activation status, macrophages have been classified as classically and alternatively activated. In turn, these different macrophage types drastically differ in the cytokines that they secrete, and consequently, their functions (8). The process of cytokine secretion is masterfully regulated by a series of interorganellar exchanges that rely on vesicular trafficking and cytoskeletal remodeling (9). Proteins regulating neurotransmitter release, notably members of the soluble *N*-ethylmaleimide-sensitive factor attachment protein receptor (SNARE) family (9, 10), and more recently synaptotagmins (Syt) (11), are pivotal for the spatiotemporal regulation of cytokine secretion. In immune cells, SNAREs and Syts have been found to regulate processes ranging from cytokine trafficking to cell migration and phagocytosis.

This review will present the functions of macrophage cytokines and, where known, summarize findings on how these cytokines are released. The types of macrophages that secrete these cytokines will also be depicted. To illustrate the importance of macrophage cytokines in health and disease, we will describe selected examples of how pathogens use cytokines to their advantage.

## The Macrophage Cytokine Portfolio

### Proinflammatory cytokines

When macrophages are exposed to inflammatory stimuli, they secrete cytokines such as tumor necrosis factor (TNF), IL-1, IL-6, IL-8, and IL-12. Although monocytes and macrophages are the main sources of these cytokines, they are also produced by activated lymphocytes, endothelial cells, and fibroblasts. Additionally, macrophages release chemokines, leukotrienes, prostaglandins, and complement. All of these molecules, in concert, may induce increased vascular permeability and recruitment of inflammatory cells. Aside from local effects, these mediators also produce systemic effects such as fever and the production of acute inflammatory response proteins. The inflammatory response is beneficial for the host when the aforementioned cytokines are produced in appropriate amounts, but toxic when produced in a deregulated fashion. For example, excessive production of IL-1β and TNF triggers an acute generalized inflammatory response characteristic of septic shock and multi-organ failure (12).

#### TNF

Tumor necrosis factor (formerly known as TNF-α) is a 185-aminoacid glycoprotein that was initially described for its ability to induce necrosis in certain tumors (13). It stimulates the acute phase of the immune response. This potent pyrogenic cytokine is one of the first to be released in response to a pathogen, and is able to exert its effects in many organs (12). As such, TNF is one of the main cytokines responsible for septic shock. In the hypothalamus, TNF stimulates the release of corticotropic releasing hormone, suppresses appetite, and induces fever. In liver, it stimulates the acute inflammatory response by elevating the synthesis of C-reactive protein and other mediators. TNF induces vasodilation and loss of vascular permeability, which is propitious for lymphocyte, neutrophil, and monocyte infiltration. It helps recruit these cells to the inflammation site by regulating chemokine release. TNF, in concert with IL-17, triggers the expression of neutrophil-attracting chemokines CXCL1, CXCL2, and CXCL5 (14) and can also augment the expression of cell adhesion molecules (15) that facilitate diapedisis. This in turn increases CXCR2-dependent neutrophil migration to the inflammation site. Being an inducer of the inflammatory response, excess amounts of TNF have been found to play pathological roles in ailments such as inflammatory bowel disease, psoriasis, rheumatoid arthritis, asthma, cancer, infectious diseases, and other auto-immune pathologies. Some of these conditions are currently co-treated with monoclonal antibodies that neutralize this cytokine (16).

In macrophages, TNF is released to the extracellular milieu via the constitutive secretion pathway, and its trafficking is the best understood of all cytokines (9, 17, 18). Details on TNF trafficking will be discussed in another article of this issue. After synthesis in the ER, the SNARE proteins Stx6, Stx7, Vtib mediate the fusion of TNF-containing vesicles from the Golgi complex with VAMP3^+^-recycling endosomes (17, 19). Thence, the Stx4/SNAP23/VAMP3 complex facilitates the passage of TNF from recycling endosomes to the cell membrane (17, 18). Rho1 and Cdc42, two proteins that govern cell shape via actin remodeling, also regulate the post-recycling endosome trafficking of TNF to the plasmalemma (20). Moreover, LPS was found to increase the expression of vesicle trafficking proteins that regulate TNF trafficking (17, 18). Finally, release of mature TNF from the plasmalemma requires cleavage of the membrane-bound precursor by the TNF-α-converting enzyme (TACE) (21). The process of phagocytosis requires extensive membrane exocytosis from several organelles that also partake in TNF secretion (7). Interestingly, it was found that TNF is not only secreted to the extracellular milieu at the plasma membrane, but also in a polarized manner at the phagocytic cup (17). This highlights an efficient and elegant strategy where macrophages can promptly release cytokines at the same time that they phagocytose microbial invaders. The importance of regulating TNF secretion implies that there exist negative regulators for its secretion. One such regulator is the recently characterized protein Syt XI, which associates to recycling endosomes and lysosomes in macrophages (11, 22). Syts constitute a group of membrane proteins that regulate vesicle docking and fusion in processes such as exocytosis (11, 23) and phagocytosis (11, 24, 25). Syts control vesicle fusion by virtue of their Ca^2+^-binding C2 domains (26). However, Syt XI cannot bind calcium and inhibits vesicle fusion (27). Upon LPS stimulation of macrophages treated with siRNA to Syt XI, more TNF and IL-6 are released. The inverse is true when Syt XI is overexpressed (11). Though the mechanism for this finding is not yet known, Syt XI likely regulates cytokine release by interacting with members of the SNARE complex. Indeed, Syt XI was found to interact with the Golgi SNARE Vti1a (28), raising the question of whether Syt XI regulates SNARE complex formation at the Golgi.

#### IL-1

Three forms of IL-1 are known: IL-1α, IL-1β and IL-1Ra. Although both IL-1α and IL-1β are strongly proinflammatory, perform many of the same functions and bind to the IL-1 receptor (IL-1R), there is only 25% aminoacid homology between them. Similarly to TNF, IL-1β is also an endogenous pyrogen that is produced and released at the early stages of the immune response to infections, lesions, and stress. Although monocytes and macrophages are the main sources of IL-1β, it is also released by NK cells, B cells, dendritic cells, fibroblasts, and epithelial cells. During inflammation, IL-1β stimulates the production of acute phase proteins from the liver and acts on the central nervous system to induce fever and prostaglandin secretion. In mast cells, IL-1β induces the release of histamine, which in turn elicits vasodilation and localized inflammation. It is also a chemoattractant for granulocytes, enhances the expansion and differentiation of CD4 T cells (29), and increases the expression of cell adhesion molecules on leukocytes and endothelial cells. Additionally, IL-1β augments the expression of genes that produce it (30). To quell the proinflammatory action of IL-1α and IL-1β, IL-1Ra competes for the same receptor. IL-1Ra is secreted via the classical secretory, though the exact mechanism is not well known. Its binding to the IL-1R does not induce the proinflammatory signaling program induced by IL-1α and IL-1β.

In stimulated macrophages, IL-1α is synthesized *de novo* and can be actively secreted (31) or passively released from apoptotic cells (32). It can also exert its effects in an intracrine fashion and act as a transcription factor (29, 30). IL-1β is synthesized as a leaderless precursor that must be cleaved by inflammasome-activated caspase-1. After activation, autophagy plays a major role in the release of this cytokine. Autophagy is a highly conserved process in eukaryotes in which the cytoplasm, aberrant, or damaged organelles are sequestered in double-membrane vesicles and released into the lysosome for breakdown and eventual recycling of resulting macromolecules (33). This process plays a crucial role in adaptation to changing environmental conditions, starvation, cellular remodeling during development, and senescence. Autophagy is characterized by the formation of double-membrane vesicles, called autophagosomes, which capture and transport cytoplasmic material to acidic compartments where material is degraded by hydrolytic enzymes (33). Autophagy has also been recognized to mediate the secretion of proteins (34) – such as IL-1β and IL-18 (35, 36) – that would otherwise not enter the classical secretory pathway due to lack of a leader peptide. In the case of IL-1β, the autophagic protein Atg5, the Golgi protein GRASP55, and Rab8a are essential for translocating IL-1β-containing cargo to the outside of the cell. In peritoneal macrophages, it has been shown that IL-1β is transported to the extracellular milieu via membrane transporters (37); knockdown of ABC transporters inhibits IL-1β secretion (38). Additionally, exocytosis of P2X7R-positive multivesicular bodies containing exosomes has also been reported to play an important role in the release of this cytokine (39). The various modes of IL-1 secretion highlight the exquisite machinery that macrophages have evolved as a means for rapidly responding to inflammatory stimuli.

#### IL-6

IL-6 is a pleiotropic cytokine that has both proinflammatory and anti-inflammatory functions that affect processes ranging from immunity to tissue repair and metabolism. It promotes differentiation of B cells into plasma cells, activates cytotoxic T cells, and regulates bone homeostasis. As with other proinflammatory cytokines, IL-6 is has been implicated in Crohn’s disease and rheumatoid arthritis (40). Similar to TNF and IL-1β, IL-6 is an endogenous pyrogen that promotes fever and the production of acute phase proteins from liver. Proinflammatory properties are elicited when IL-6 signals in trans via soluble IL-6 receptors binding to gp130, which is ubiquitous in all cells. Inhibition of trans signaling via gp130 blockade in murine sepsis models rescues mice from widespread inflammation and death (41). IL-6 trans signaling also leads to recruitment of monocytes to the inflammation site (42), promotes the maintenance of Th17 cells, and inhibits T cell apoptosis and development of Tregs (43). In contrast, anti-inflammatory properties are elicited when IL-6 signals through the classical pathway, which occurs via the IL-6 receptor that only few cells express. The anti-inflammatory properties of IL-6 are illustrated by IL-6^−/−^ mice, which exhibit hepatosteatosis, insulin resistance, and liver inflammation (44). IL-6 classic signaling also mediates apoptosis inhibition and the regeneration of intestinal epithelial cells (43).

IL-6 is a soluble cytokine that is synthesized in the ER and, unlike TNF, is not processed as a membrane-bound precursor. Upon stimulation of macrophages with LPS, IL-6 starts accumulating in the Golgi after 4 h of stimulation (45). From the Golgi, IL-6 exits in tubulovesicular carriers that may also contain TNF. Golgi-derived vesicles then fuse with VAMP3-positive recycling endosomes. Three-dimensional reconstruction of fluorescence images showed that recycling endosomes can harbor both TNF and IL-6, albeit both occupy different subcompartments (45). The post-Golgi trafficking of IL-6 follows a route that is also dependent on Stx6 and Vti1b, which form a complex with cognate SNARE VAMP3 at recycling endosomes (17, 18). Knockdown and overexpression of these SNAREs decreases and augments IL-6 release, respectively (45). Syt XI may be negatively modulating the secretion of this cytokine by regulating the formation of these SNARE complexes (11, 28). Unlike TNF, IL-6 is not secreted at the phagocytic cup (45).

#### IL-12

IL-12 is produced primarily by monocytes, macrophages, and other antigen-presenting cells; it is essential for fighting infectious diseases and cancer. IL-12 is a heterodimeric cytokine comprised of the p35 and p40 subunits, which come together after their synthesis. Deletions within the p40 gene have been observed in patients suffering from concurrent multiple bacterial infections (46, 47). IL-12 promotes cell-mediated immunity via stimulation of Th1 cells. It synergizes with TNF and other proinflammatory cytokines in stimulating IFN-γ production, as well as the cytotoxicity of NK and CD8 T cells (48). IL-12 can also inhibit angiogenesis through IFN-γ-mediated upregulation of the anti-angiogenic chemokine CXCL10. The involvement of this cytokine in these processes has made it a target in both auto-immune pathologies and cancer (46, 47). After protein synthesis, both p40 and p35 subunits associate at the ER, where they undergo subsequent glycosylation steps prior to being released at the cell membrane (49). Although the precise post-Golgi trafficking mechanisms in macrophages are not known, the release route is likely to resemble that of TNF and IL-6 (9). Data from neutrophils localized the SNAREs VAMP2, VAMP7, Stx2, Stx6, and SNAP23 in the granules that contain and secrete IL-12 (50, 51). Although macrophages do not possess secretory granules, IL-12 release from these cells may involve some of the same SNARE complexes. Furthermore, IL-12 is secreted in a polarized manner from lymphocytes; this process is dependent on Cdc42 (52), which also regulates release of TNF to the plasma membrane. This raises the interesting prospect that IL-12 may be released in a polarized fashion, along with TNF (17), at nascent macrophage phagosomes.

#### IL-18

IL-18 is a member of the IL-1 family and also an inducer of IFN-γ production. It synergizes with IL-12 to activate T cells and NK cells. Albeit the fact that IL-18 signals similarly to IL-1β, IL-18 is not a pyrogen, and can even attenuate IL-1β-induced fever (53). Lack of fever induction may be explained by the fact that IL-18 signals through the MAPK p38 pathway instead of the NF-κB pathway, which is used by IL-1β (54). IL-18 trafficking is similar to that of IL-1β, with secretory autophagy also playing a major role in its release (35, 36).

#### IL-23

IL-23 is also an IFN-γ inducer and T cell activator that is involved in a variety of diseases ranging from psoriasis to schizophrenia (47). It is similar to IL-12 in that both induce inflammation. Moreover, both IL-12 and IL-23 share the IL-12p40 subunit and thus have similar signaling pathways. In contrast to IL-12, IL-23 augments IL-10 release and induces IL-17 synthesis by activated naïve T cells (55).

#### IL-27

IL-27 is a member of the IL-12 family, and is composed of subunits p28 and Epstein–Barr virus-induced gene 3. Similar to TNF, it is produced early in monocytes and macrophages stimulated with LPS and IFN-γ. Knockout of its receptor ensues in increased susceptibility of mice to bacterial and parasitic infections due to impaired IFN-γ production (56). In addition to favoring the differentiation of naïve T cells to Th1 cells via IFN-γ induction, IL-27 can also inhibit the differentiation of Th17 cells (57). IL-27 also has anti-inflammatory properties, which are exemplified by the fact that IL-27 receptor-deficient mice are more susceptible to auto-immune encephalomyelitis, which correlates with increased levels of Th17 cells (55). The fact that this cytokine has selective inflammatory and anti-inflammatory properties supports the concept that the inflammatory response is prompt, but also carefully calibrated to avoid damage to the host.

### Anti-inflammatory cytokines

#### IL-10

Inflammation is tightly regulated by multiple inhibitors and antagonists. IL-10 is a 35 kD cytokine identified in 1989, and is produced by activated macrophages, B cells, and T cells (58). Its main activities concern the suppression of macrophage activation and production of TNF, IL-1β, IL-6, IL-8, IL-12, and GM-CSF (59). IL-10 suppresses MHC-II expression in activated macrophages and is thus a potent inhibitor of antigen presentation (60). Of particular interest is that IL-10 inhibits the production of IFN-γ by Th1 and NK cells, and induces the growth, differentiation, and secretion of IgGs by B cells (61, 62). Macrophages themselves are affected by IL-10 in that exposure to this cytokine lowers their microbicidal activity and diminishes their capacity to respond to IFN-γ (63, 64). Experiments in murine models have shown that blocking or neutralizing IL-10 leads to increased levels of TNF and IL-6; on the contrary, exogenous IL-10 improves survival and reduces the levels of inflammatory cytokines (65). It has been observed that reduced levels of IL-10 favor the development of gastrointestinal pathologies such as inflammatory bowel disease (65). Recombinant IL-10 has indeed been effective in the treatment of some of these diseases.

The mechanism of IL-10 trafficking and release resembles that of TNF and IL-6 (66). IL-10 traffics from Golgi tubular carriers associated with p230/golgin-245 along with TNF-containing vesicles, or in golgin-97-associated tubules. The Golgi-associated p110δ isoform of PI3K was also found to be a positive regulator of IL-10 release. From the Golgi, IL-10-containing vesicles move to recycling endosomes, where VAMP3 and Rab11 then modulate the transit of this cytokine – and of TNF and IL-6 – to the cell surface. Independent of recycling endosomes, IL-10 was also observed to exit directly from the Golgi to the cell surface in apoE-labeled vesicles (66).

#### TGF-β

Together with IL-10, TGF-β is another powerful anti-inflammatory cytokine that acts on many target cells and tones down the inflammatory effects of TNF, IL-1β, IL-2, and IL-12, etc. (61, 67, 68). TGF-β is a potent suppressor of both Th1 and Th2 cells, but foments the maintenance and function of Tregs (67, 69). The importance of TGF-β in the immune system is highlighted by the fact that mice lacking the TGF-β1 isoform, which is predominant in cells of the immune system, develop severe multi-organ inflammation and die by week 4 (70). TGF-β is also implicated in hematopoiesis and has a crucial role in embryogenesis, tissue regeneration, and cell proliferation and differentiation.

Transforming growth factor beta is synthesized as a precursor and is directed to the ER by virtue of its signal peptide. Cleavage by the endoprotease furin, which can happen at the ER or in the extracellular environment, is required for activation of this cytokine (70). Although the secretory mechanism of this cytokine has not been explored, it is possible that it follows a post-Golgi pathway similar to that of TNF, IL-6, or IL-10.

### Chemokines

Chemokines are a special family of heparin-binding cytokines that are able to guide cellular migration in a process known as chemotaxis. Cells that are attracted by chemokines migrate toward the source of that chemokine. During immune surveillance, chemokines play a crucial role in guiding cells of the immune system to where they are needed (71). Some chemokines also play a role during development by promoting angiogenesis, or by guiding cells to tissues that provide critical signals for the cell’s differentiation. In the inflammatory response, chemokines are released by a wide variety of cells involved in both innate and adaptive immunity (71). As already mentioned, chemokine release is often induced by proinflammatory cytokines such as TNF, IL-6, and IL-1β. Below is a description of the main chemokines released by macrophages.

#### CXCL1 and CXCL2 (MIP-2α)

CXCL1 and CXCL2 (also known as macrophage inflammatory protein 2-α, MIP) share 90% amino acid similarity and are secreted by monocytes and macrophages to recruit neutrophils and hematopoietic stem cells (72, 73). Both chemokines are angiogenic and may promote the development of tumors such as melanomas (74).

#### CCL5 (RANTES)

CCL5, or the regulated upon activation normal T cell expressed and secreted (RANTES), is an inflammatory chemoattractant for T cells, basophils, eosinophils, and dendritic cells to the site of inflammation (75). Aside from this role, it can also mediate the activation of NK cells into chemokine-activated killers (CHAK) (76). Similar to CXCL1 and 2, it promotes tumorigenesis and metastasis (74). CCL5 is synthesized in the ER and traffics to the Golgi complex before being exported outside of the cell. The secretory carrier membrane protein (SCAMP)5, a recycling endosome-associated protein, governs post-Golgi trafficking of CCL5 to the plasmalemma. Stimulation of macrophages with ionomycin induces SCAMP5 translocation to the plasma membrane, where it colocalizes and interacts with Syt I and II, which in turn mediate interactions with various SNAREs (77).

#### CXCL8 (IL-8)

CXCL8 is a potent chemoattractant for neutrophils, in which it also induces degranulation and morphological changes (78, 79). Since macrophages are some of the first cells to respond to an antigen, they are likely the first cells to release CXCL8. Other cells such as keratinocytes, endothelial cells, eosinophils, and basophils also respond to this chemokine. The importance of IL-8 has made this chemokine important in inflammatory diseases such as psoriasis, Crohn’s disease, and cancer (80, 81).

#### CXCL9 (MIG)

CXCL9, also known as monokine induced by gamma interferon (MIG), is a strong T cell chemoattractant to the site of inflammation (71, 82). It mediates cell recruitment necessary for inflammation and repair of tissue damage. CXCL9 also inhibits neovascularization (83) and has anti-tumor and anti-metastatic effects (74).

#### CXCL10 (IP-10)

CXCL10, or interferon gamma-induced protein 10, is secreted not only by monocytes and macrophages, but also by fibroblasts and endothelial cells (83). It serves to attract T cells, NK cells, dendritic cells (84), and also has potent anti-cancer activity.

#### CXCL11 (IP-9)

Similar to CXCL9 and CXCL10, CXCL11 is interferon-inducible and also mediates T cell recruitment, although more potently than CXCL9 and CXCL10 (85). It also inhibits angiogenesis and tumor formation (74).

## Alternatively Activated Macrophages and Their Cytokines

**Figure 1:**
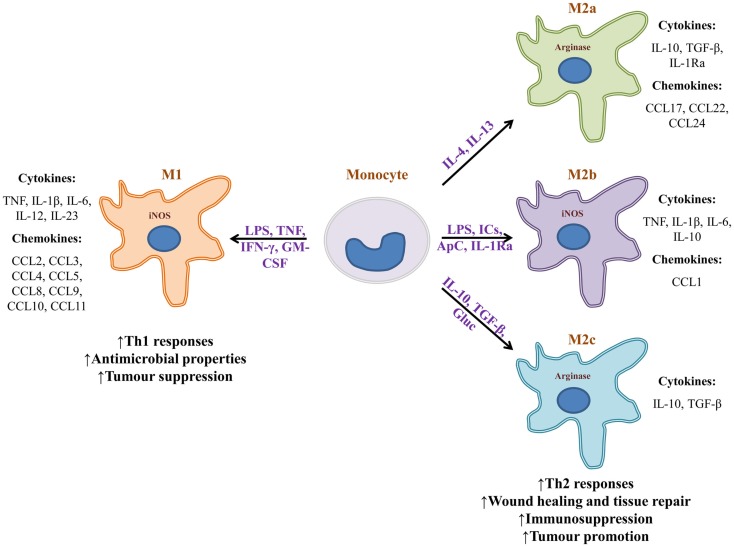
**Monocytes can become phenotypically distinct macrophages**. Upon encountering different stimuli, monocytes turn into highly microbicidal (M1), or into immunosuppressive macrophages (M2). Stimuli can range from microbial substances to biochemical signals provided by the microenvironment of a given tissue. Many of the cytokines that bias macrophage phenotype are provided by surrounding lymphocytes or other non-immune cells. Macrophage subtypes release a vastly different array of cytokines and chemokines that can either promote inflammation and sometimes tissue destruction, or wound healing and tissue repair. M1 macrophages are known to be tumor suppressive whereas M2 macrophages generally promote tumorigenesis. It is important to note that macrophage bias is a spectrum and is reversible. IC, immune complexes; ApC, apoptotic cells; Gluc, glucocorticoids.

The microenvironment in which a macrophage is found provides it with diverse signals that divergently bias the macrophage’s phenotype toward “classically activated” (M1) or “alternatively activated” (M2a, M2b, or M2c) (Figure 1) (55). Polarization signals may be apoptotic cells, hormones, immune complexes, or cytokines provided by lymphocytes or other cells. Exposure of naïve monocytes or recruited macrophages to the Th1 cytokine IFN-γ, TNF, or LPS, promotes M1 development. Those macrophages in turn secrete proinflammatory cytokines TNF, IL-1β, IL-6, IL-12, IL-23, and promote the development of Th1 lymphocytes. In addition, M1 macrophages secrete high levels of reactive oxygen species (ROS) and reactive nitrogen species (RNS), produce and secrete iNOS, and promote the metabolism of arginine into nitric oxide and citrulline. As a result, M1 macrophages foster a highly microbicidal environment, and have a role in mediating the destruction of pathogens and tumor cells. M1-derived chemokines help recruit NK and Th1 cells. In stark contrast, exposure or treatment of monocytes with IL-4 and IL-13 polarizes these cells toward an M2a phenotype (8, 86). Those macrophages express a series of chemokines that promote the accrual of Th2 cells, eosinophils, and basophils. M2b macrophages are induced by a combination of LPS, immune complexes, apoptotic cells, and IL-1Ra. They secrete high levels of IL-10, but also proinflammatory cytokines TNF and IL-6 and express iNOS. Through chemokine production, M2b macrophages also promote recruitment of eosinophils and Tregs that foster a Th2 response. M2c macrophages are induced by a combination of IL-10, TGF-β, and glucocorticoids. In turn, those macrophages secrete IL-10 and TGF-β, both of which are immunosuppressive cytokines that promote the development of Th2 lymphocytes and Tregs. They also express high levels of arginase and promote tissue regeneration and angiogenesis (8, 87). The capacity of M2c macrophages to induce Tregs makes them more effective than M2a macrophages at protecting organs from injury caused by inflammatory infiltrates (88). Macrophage bias is reversible. For example, if an M1 macrophage is given apoptotic cells, it may transform into an M2 macrophage.

The characteristics of M1 and M2 macrophages have implicated them in the development of infectious disease and cancer. For example, helminth-derived molecules can strongly bias macrophages toward an M2 phenotype. The cytokines and associated Th2 response that ensues promote immunosuppression and parasite survival (89). In cancer, tumor-associated macrophages (TAMs) have been known to either promote or hinder neoplasia (8, 90). In colorectal cancer, TAMs are inflammatory and promote the development of a Th1 response (91). In contrast, many other neoplasms are associated with M2-like TAMs that secrete immunosuppressive cytokines that promote tumor growth and metastasis (8, 90). TAMs may aid tumor growth by facilitating the chemotaxis of Th2 and Treg cells, and by promoting angiogenesis and lymphoangiogenesis via production of VEGF, VEGF-C and -D, PDGF, and TGF-β (92). Additionally, TAMs secrete MMP9, a matrix metalloprotease that promotes tumor growth and spread. Importantly, TAMs induce immunosuppression via release of IL-10 and TGF-β, both of which inhibit the development of cytotoxic T cells and NK cells, and may fuel the appearance of more M2-like TAMs at the tumor site (8, 67, 90). The contribution of alternatively activated macrophages and their cytokines to disease has made them a target for immunotherapies that seek to alter the phenotypic bias of macrophage populations. For instance, helminth-derived molecules could be used to alter the proinflammatory cytokine profile of colitis-associated macrophages (89).

## How Do Pathogens Disrupt Cytokine Secretion from Macrophages?

The evolutionary race that has taken place over millions of years among pathogens and their hosts has given rise to a multitude of adaptations that have allowed these pathogens to resist the defenses mounted by their hosts. Several of these adaptations endow pathogens to evade the immune system in order to survive destruction and thrive. Both intracellular and extracellular parasites have evolved mechanisms to not only avoid or survive the immune response, but also to use it for their own benefit (93, 94). Upregulating or downregulating the production and release of macrophage cytokines can have profound effects on the immune response. A variety of pathogenicity factors target these important molecules of the immune system. The following examples describe how certain pathogens, depending on their needs, deregulate cytokine secretion to aid in their survival and dissemination.

### *Mycobacterium ulcerans* uses mycolactone to inhibit cytokine production

**Figure 2:**
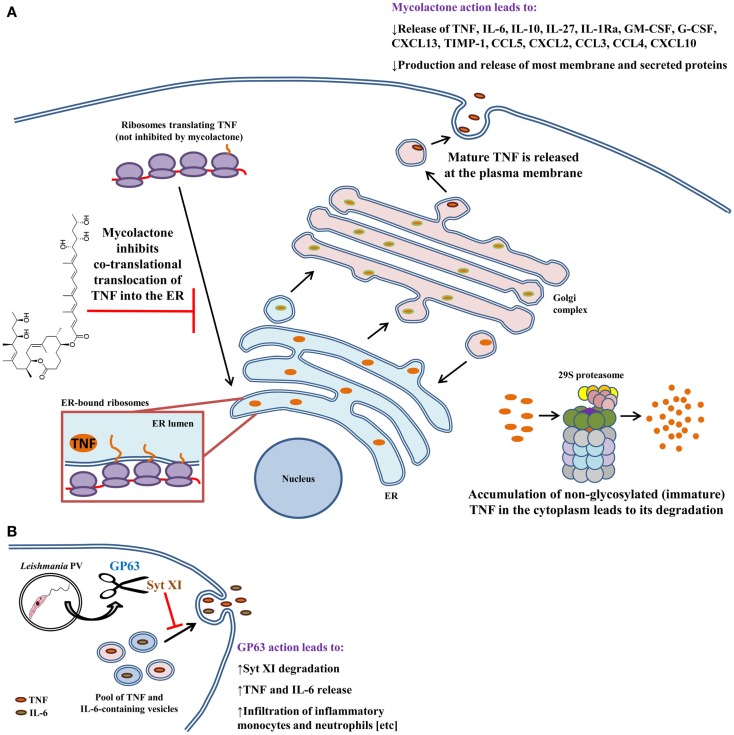
**Modulation of macrophage cytokine secretion by *Mycobacterium ulcerans* bacteria and *Leishmania* promastigotes**. Disruption of cytokine release has evolved as an effective means by which several pathogens contravene the immune response. **(A)** *M. ulcerans* employs mycolactone to sabotage the immune response by inhibiting the secretion of more than 17 cytokines, chemokines, and inflammatory mediators. TNF, as well as other cytokines and chemokines, undergo post-translational modifications in the ER and Golgi prior to being shepherded outside of the macrophage. Mycolactone hampers delivery of TNF into the ER. As a consequence, immature protein that accumulates in the cytoplasm is eventually degraded by the proteasome. **(B)** Unlike *M. ulcerans, Leishmania* promastigotes trigger the release of TNF and IL-6 from infected macrophages via GP63-mediated degradation of Syt XI (a negative regulator of cytokine release). *In vivo*, GP63 also facilitates the infiltration of inflammatory monocytes and neutrophils to the infection site. Both of these phagocytes are infection targets for *Leishmania* and aid in establishing infection. These findings can be explained by the fact that TNF and IL-6 mediate phagocyte infiltration by upregulating the expression of adhesion molecules and chemokines. Arrows indicate multiple steps and drawings are not to scale.

Mycobacteria are intracellular pathogens that cause a variety of human diseases that are difficult to treat. Due to their particular cell wall, these bacteria are very resistant to antibiotics and innate host defenses. *M. ulcerans*, the causative agent of the Buruli ulcer, induces deep necrotizing ulcers that are often ironically painless (95). Lesions can cause incapacitation, disfigurement, and severe deformities (95). The disease is the third-most common mycobacterial infection and affects areas of the world with hot and humid climates. *M. ulcerans* produces a macrolide toxin called mycolactone that is highly cytotoxic and immunosuppressive (96, 97). It causes broad tissue damage in the absence of an acute inflammatory response. Injection of mycolactone alone can induce lesions similar to those caused by infection (96). In contrast to other mycobacterial infections, *M. ulcerans* is found mostly extracellularly. This may be explained by the fact that mycolactone inhibits phagocytosis and hampers phagolysosomal maturation in macrophages (98, 99). In addition, mycolactone contributes to immunosuppression by hampering the production of several cytokines and chemokines from macrophages (Figure 2A) (99–101); mycolactone is effective at dampening the production of LPS-induced mediators. Although the mechanism for these findings was unknown, data from multiple studies suggested that inhibition was at the post-transcriptional level. Indeed, Hall et al. found that mycolactone does not cause gross changes in translation, with proinflammatory mRNAs being actively translated (102). That finding prompted the investigators to check whether TNF was being translocated to the ER for processing. Interestingly, inhibiting the 29S proteasome showed that non-glycosylated TNF accumulates in the cytoplasm of mycolactone-treated macrophages, indicating that this causes the failure in TNF secretion. To show that TNF was not being translocated into the ER lumen, Hall et al. performed *in vitro* translation assays with ER-containing membranes to study whether TNF was being protected from proteinase K degradation (102). In the presence of mycolactone, TNF is not protected from proteinase K digestion, indicating that this cytokine does not translocate into the ER under these conditions. These effects were found not to be due to mycolactone disrupting ER membrane integrity or due to induction of ER-associated degradation pathways. It would be interesting to investigate whether mycolactone can physically block the channel activity of the Sec61, or that of other ER translocons. These findings were made more general by showing that – in many cell types – mycolactone was inhibiting the translocation of several secreted and membrane proteins into the ER. Importantly, mycolactone blocked the release of several cytokines, chemokines, and other inflammatory mediators from LPS-activated macrophages (102). Quenching cytokine production in this way can thus severely obstruct the development of the immune response and promote the survival of *M. ulcerans*.

### *Leishmania* promastigotes employ GP63 to augment TNF and IL-6 release

Protozoa of the *Leishmania* genus are parasites of phagocytic cells, especially macrophages. Depending on the species, *Leishmania* can cause self-healing cutaneous lesions (e.g., *L. tropica, L. major, L. mexicana*, and *L. pifanoi*), disfiguring mucocutaneous disease (e.g., *L. braziliensis* and *L. guyanensis*), or severe visceral illness (e.g., *L. donovani* and *L. Infantum chagasi*). Mucocutaneous and visceral disease can be lethal if untreated, but most deaths are attributable to visceral leishmaniasis (103). *Leishmania* has a digenetic lifecycle. Promastigotes are elongated and have a flagellum that allows them to move in extracellular environments. Dividing procyclic promastigotes develop in the gut of infected sandflies, where they transform into infectious non-dividing metacyclic promastigotes that can be ejected upon the sandfly’s next blood meal (104). Once inside the host, metacyclic promastigotes are phagocytosed by neutrophils or by macrophages. *Leishmania* promastigotes are able to cripple the microbicidal power of the phagosome, rendering it a propitious parasitophorous vacuole (PV) for the parasite (105, 106). Within PVs, promastigotes differentiate into amastigotes, which are the non-flagellated intracellular form of the parasite. Amastigotes replicate inside macrophages, and when these apoptose, surrounding macrophages uptake the amastigote cargo (107), eventually propagating the infection. The *Leishmania* lifecycle is perpetuated when free amastigotes and amastigote-containing phagocytes are taken up by sandflies that bite infected hosts. The GP63 zinc metalloprotease is a multifaceted *Leishmania* pathogenicity factor and is also one of the most abundant molecules on the surface of promastigotes (105, 108, 109). In infected macrophages, GP63 impairs antigen cross-presentation (110), stalls transcription and translation, and deactivates several microbicidal pathways (111–114). Additionally, GP63 hampers lipid metabolism in liver, and helps the parasite evade complement-mediated lysis and avoid killing by NK cells (108, 115, 116). Of particular note is the capacity of GP63 to cleave members of the SNARE complex (105), which raises the possibility that GP63 may cleave other membrane fusion regulators. Earlier studies found that *Leishmania* promastigotes of certain species were able to induce the release of TNF and IL-6 (117–121) following their engulfment by macrophages. However, the mechanisms for this induction were not known. Hence, Arango Duque et al. hypothesized that Syt XI, a negative regulator of cytokine secretion (11), was targeted by *Leishmania* (Figure 2B) (22). Infection of macrophages with GP63^+/+^ or GP63^−/−^ parasites revealed that Syt XI is degraded by GP63, leading to the release of TNF and IL-6. Moreover, cytokine release by infected macrophages positively correlated with the GP63 content of different *Leishmania* species. To highlight the relevance of these findings in an *in vivo* setting, it was demonstrated that intraperitoneal injection of GP63-expressing promastigotes induces TNF and IL-6 release 4 h after inoculation. As already described, these cytokines induce adhesion factor expression and chemokine release (14, 15, 42, 122, 123). Interestingly, it was observed that GP63 also promotes the infiltration of neutrophils and inflammatory monocytes early during infection. Future research will reveal whether phagocyte recruitment is dependent on GP63-mediated cleavage of Syt XI *in vivo*. It will also be interesting to research whether Syt XI is targeted by other pathogens. The involvement of GP63 in cytokine secretion and phagocyte recruitment can aid in the establishment of infection. Infection of recruited inflammatory monocytes and resident macrophages can induce IL-10 secretion, which fosters the immunosuppressive environment observed in chronic infection (124, 125). Infection of inflammatory monocytes (126) may also turn them into arginase-expressing alternatively activated macrophages that trigger the differentiation of naïve CD4 T cells into FoxP3^+^ cells (86), which are immunosuppressive in leishmaniasis (127, 128). Overall, those findings underline the importance of proinflammatory cytokines and phagocytes at the early stages of *Leishmania* infection (22).

## Conflict of Interest Statement

The authors declare that the research was conducted in the absence of any commercial or financial relationships that could be construed as a potential conflict of interest.
